# Variants in the 5′UTR reduce *SHOX* expression and contribute to *SHOX* haploinsufficiency

**DOI:** 10.1038/s41431-020-0676-y

**Published:** 2020-07-09

**Authors:** Deepak Babu, Silvia Vannelli, Antonella Fanelli, Simona Mellone, Ave Maria Baffico, Lucia Corrado, Wael Al Essa, Anna Grandone, Simonetta Bellone, Alice Monzani, Giulia Vinci, Luisa De Sanctis, Liborio Stuppia, Flavia Prodam, Mara Giordano

**Affiliations:** 1 Laboratorio di Genetica Umana, Dipartimento di Scienze della Salute, Università del Piemonte Orientale, Novara, Italy; 2 Endocrinologia Pediatrica, Dipartimento di Pediatria e Specialità Pediatriche, Ospedale Regina Margherita, Citta della Salute e della Scienza, Torino, Italy; 3 Laboratorio di Genetica e Biologia Molecolare, S.C.D.U. Biochimica Clinica AOU “Maggiore della Carità”, Novara, Italy; 4 Laboratorio di Genetica Umana, IRCCS G.Gaslini, Genova, Italy; 5 Dipartimento della Donna, del Bambino, di Chirurgia Generale e Specialistica, Università degli Studi della Campania “L. Vanvitelli”, Napoli, Italy; 6 Dipartimento di Scienze della Salute, Università del Piemonte Orientale, Divisione di Pediatria, AOU “Maggiore della Carità”, Novara, Italy; 7 Dipartimento di Scienze Psicologiche della Salute e del Territorio, Università degli Studi G. D’Annunzio, Chieti, Italy; 8 Center for Advanced Sciences and Technologies (CAST), Università degli Studi G. D’Annunzio, Chieti, Italy; 9 Dipartimento di Scienze della Salute, Università del Piemonte Orientale, Divisione di Endocrinologia, AOU “Maggiore della Carità”, Novara, Italy

**Keywords:** Mutation, RNA splicing

## Abstract

*SHOX* haploinsufficiency causes 70–90% of Léri-Weill dyschondrosteosis (LWD) and 2–10% of idiopathic short stature (ISS). Deletions removing the entire gene or enhancers and point mutations in the coding region represent a well-established cause of haploinsufficiency. During diagnostic genetic testing on ISS/LWD patients, in addition to classic *SHOX* defects, five 5′UTR variants (c.-58G > T, c.-55C > T, c.-51G > A, c.-19G > A, and c.-9del), were detected whose pathogenetic role was unclear and were thus classified as VUS (Variants of Uncertain Significance). The purpose of the present study was to investigate the role of these noncoding variations in *SHOX* haploinsufficiency. The variants were tested for their ability to interfere with correct gene expression of a regulated reporter gene (luciferase assay). The negative effect on the mRNA splicing predicted in silico for c.-19G > A was assayed in vitro through a minigene splicing assay. The luciferase assay showed that c.-51G > A, c.-19G > A, and c.-9del significantly reduce luciferase activity by 60, 35, and 40% at the homozygous state. Quantification of the luciferase mRNA showed that c.-51G > A and c.-9del might interfere with the correct SHOX expression mainly at the post-transcriptional level. The exon trapping assay demonstrated that c.-19G > A determines the creation of a new branch site causing an aberrant mRNA splicing. In conclusion, this study allowed us to reclassify two of the 5′UTR variants identified during SHOX diagnostic screening as likely pathogenic, one remains as a VUS, and two as likely benign variants. This analysis for the first time expands the spectrum of the genetic causes of *SHOX* haploinsufficiency to noncoding variations in the 5′UTR.

## Introduction

The short-stature homeobox gene (*SHOX*), is located within the pseudoautosomal region 1 (PAR1) on Xp22.33/Yp11.32 and encodes a transcription factor that regulates chondrocyte proliferation and differentiation in the growth plate [1]. The long-range transcriptional regulation of *SHOX* is controlled by seven cis-acting conserved noncoding elements (CNEs), residing both upstream and downstream of the gene that exhibits enhancer function in chicken limb buds and neural tubes [2].

*SHOX* haploinsufficiency (the loss of function of one *SHOX* allele), results in a wide variety of short stature phenotypes. Molecular defects in *SHOX* were frequently reported to be responsible for short stature in patients with Leri-Weill syndrome (LWD; MIM #127300), characterized by skeletal dysplasia with disproportionate short stature, mesomelia (i.e., shortening of the middle parts of the limbs) and characteristic Madelung wrist deformity [3–5]. *SHOX* mutations were also identified as the cause of growth retardation in 2–10% of individuals with idiopathic short stature (ISS; MIM#300582) [3, 6–11]. On the other hand biallelic loss of *SHOX* expression results in an extreme phenotype of osteodysplasia called Langer mesomelic dysplasia (MIM #249700), which is characterized by extreme short stature, severe shortening or aplasia of the ulna and fibula, and both thickening and curvature of the radius and tibia [12].

Individuals carrying *SHOX* alterations exhibit considerable phenotypic heterogeneity, and in several cases the proband’s relatives carrying the causative variants are apparently asymptomatic with normal height.

Approximately 75% of the *SHOX* defects are represented by deletions of different sizes encompassing the whole gene and/or the CNEs, whereas intragenic microdeletions and point mutations occur in the rest of the cases [7, 13]. The enhancer deletions generate phenotypes indistinguishable from single nucleotide variants and deletions affecting the *SHOX* coding region [14] and identical alterations can determine either LWS or ISS. Rarely, duplications of *SHOX* and/or its enhancers have been reported in patients [15–18] although their causative role remains contradictory.

The wide spectrum of phenotypes is likely the result of different degrees of SHOX deficiency attributable to variations that differentially alter the expression levels in combination with different genetic backgrounds.

The correct SHOX function is strictly dose-dependent, as demonstrated by the observation that ~25–50% of the causative deletions encompass only the cis-acting enhancer, leaving the coding region intact [13, 19, 20]. It is thus conceivable that variants in regulatory regions other than the long-range cis-acting enhancers might lead to some degree of SHOX deficiency in ISS/LWS.

The short-range transcription of *SHOX* is directed by two alternative promoters, P_1_ (upstream promoter) and P_2_ (intragenic promoter), generating transcripts with a 5′UTR of different lengths encoding the same protein. The molecular mechanism underlying the preferential usage of one promoter over the other remains unknown. There is evidence that the transcripts generated from P_1_ exhibit significant translation inhibitory effects due to seven AUG codons upstream of the main open reading frame [21] while the mRNAs from P_2_ are translated with higher efficiency, probably in situations of immediate need of high SHOX amounts. Using several reporter constructs, Blaschke et al. [21] narrowed down the intragenic promoter P_2_ to a region of 300 bp upstream of the AUG start codon. This region contains a canonical TATA box (c.-137) and a CAAT box (c.-257), indicating the core promoter characteristics of P_2._

During the diagnostic screening of *SHOX* performed in the last 8 years on ISS/LWD patients, we identified several individuals carrying different noncoding variants in exon 2 within the 5′UTR of both the transcripts that were classified as VUS (Variants of Uncertain Significance).

This work aimed to investigate the pathogenic role of these variants by testing their ability to interfere with the correct gene expression and consequently to alter the dosage of the SHOX protein. Our results showed that these noncoding variants might be responsible for *SHOX* haploinsufficiency either through a reduced gene expression or by affecting the correct RNA splicing.

## Subjects and methods

### Subjects

The *SHOX* diagnostic testing included 1036 unrelated individuals recruited from multiple Italian centers. Informed written consent was obtained from all the patients or their parents. The patients presented with different phenotypes ranging from ISS to extreme disproportionate short stature to the most severe form of LWD.

For every patient, height, weight, and BMI were stratified according to the Italian growth charts [22]. Measurements of standing height, sitting height (measured from the highest point of the head to the sitting surface) [23], arm span (length from the fingertips of one hand to the other with the arms raised parallel to the ground) [23], and growth velocity (difference of mean heights obtained from two consecutive visits, divided by the time between the visits) were recorded. The stature was considered short when corresponding to <-2 SDS for age, sex, and population or stature below the genetic target according to Tanner’s method [24].

**Fig. 1:**
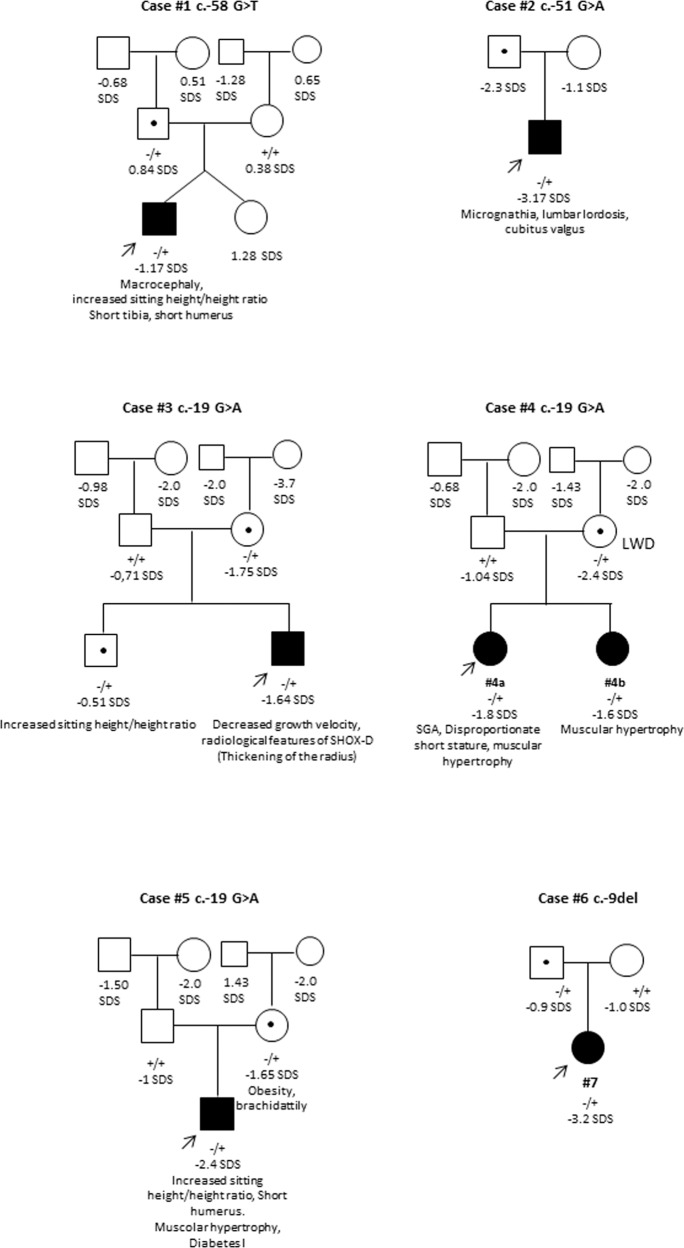
Pedigrees of patients carrying the 5′UTR variants. The proband of each family is indicated by an arrow. The variant carried by the proband is reported below each pedigree. The genotypes of all the family members that were available are reported (+ = wild-type allele; − = variant allele). The filled symbols indicate individuals with clinical feature suggestive of SHOX haploinsufficiency. The height SDS and phenotypes are reported under each symbols. LWD Leri-Weill dyschondrosteosis.

Particular attention was given to clinical sign characteristic of *SHOX* haploinsufficiency such as: cubitus valgus, short forearm, bowing of the forearm, muscular hypertrophy, dislocation of ulna and BMI percentile. A combination of these signs together with sitting height/height ratio and arm span/height ratio was used retrospectively to calculate the Rappold Score [7] (RS) that has is considered as an indicator of SHOX deficiency when ≥4, also in absence of short stature that is not considered for the score calculation. All the patients carrying the 5′UTR variants had either a RS ≥4 or severe short stature (Table 1). The clinical characteristic of these patients and their pedigrees are reported in Table 1 and Fig. 1, respectively. The height growth charts for the index cases have been included in the supplementary information (Supplementary Figure 1a–f).

**Table 1:** Clinical characteristics of the patients carrying the *SHOX* 5′UTR variants.

Patient	Variation	Sex	Age at diagnosis	Height (cm)	Height SDS	Gestational age	Weight at birth (g)	Length at birth (cm)	Bone age (years)	Growth velocity (cm/year)	Growth velocity SDS	Target height SDS	Weight percentile	BMI percentile	Sitting height/height	Arm span/height	Cubitus Valgus	Short forearm	Curved forearm	Muscular hypertrophy	Ulnar dislocation (at the elbow)	Rappold score
1	c.-58 G > T	M	10.3	131.1	−1.17	39	2920	50	8	5	−0.13	0.67	25–50	>50	0.556	na	No	No	No	No	No	6
2	c.-51 G > A	M	12.2	125	−3.17	40	2640	47	12.7	na	na	−1.9	<3	15	0.49	1.05	Yes	No	No	No	No	2
3	c.-19 G > A	M	10.7	130.4	−1.64	39	3850	51.5	9.3	4.6	−0.67	−1.17	50	50–85	0.537	0.998	No	No	No	No	No	4
4.a	c.-19 G > A	F	9.6	124.2	−1.8	39	3130	45.4	9.2	4.3	−1.41	−2.10	25–50	85–97	0.553	0.998	No	No	No	Yes	No	9
4.b	c.-19 G > A	F	7.7	116.8	−1.6	40	3490	48	na	5.2	−0.61	−2.10	25	50–85	0.55	0.990	No	No	No	Yes	No	9
5	c.-19 G > A	M	16.4	155	−2.4	39	4760	53	17	na	na	−1.71	25	50–85	0.57	1	No	No	No	Yes	No	9
6	c.-9del	F	11.6	126	−3.2	40	2770	49	10.8	8.4	0.1	−2.0	1	1	0.54	0.99	No	No	No	No	No	0

*na* not available.

A panel of 759 normal statured (−0.5 ≥ SDS ≤ 2.2) healthy individuals matched for sex and geographical origin was also screened for the exon 2 variations.

### Genetic analysis of *SHOX*

Genomic DNA was extracted from lymphocytes using a QIAamp DNA Kit (Qiagen, Hilden, Germany). The entire *SHOX* coding region (exon 2–exon 6a/6b), 5′UTR (exon 1–exon 2) and intron–exon boundaries were amplified by PCR (Supplementary Table 1). The PCR products were visualized on a 2% agarose gel and purified using ExoSAP-IT enzymatic PCR clean-up system (Thermo Fisher). The purified products were then sequenced with Big Dye Terminator Kit (Applied Biosystems, Foster City, CA) and automatic sequencer ABI PRISM 3100 Genetic Analyzer (Applied Biosystems).

Search for deletions/duplications of the single exons, of the entire gene, and of the upstream and downstream enhancers was performed by an MLPA assay using an MLPA Commercial Kit (SALSA MLPA Kit P018-G1 SHOX; MRC-Holland, Amsterdam, Netherlands) following the manufacturer’s instructions.

### Nomenclature

**Fig. 2:**
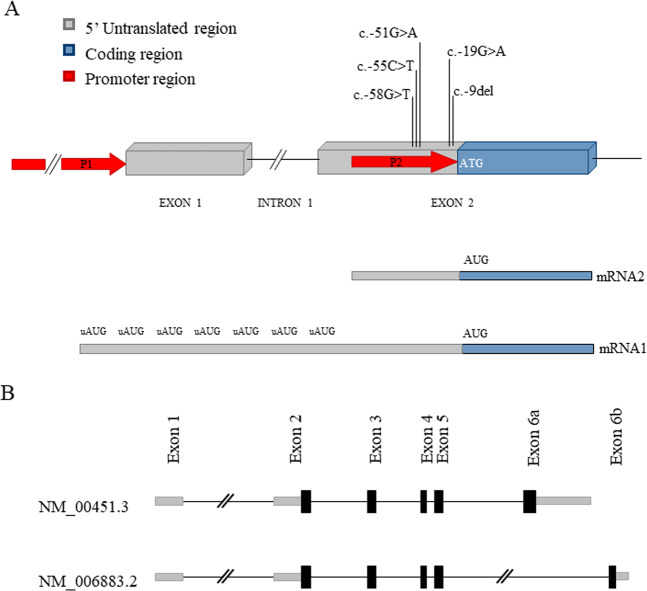
Structure of *SHOX*proximal promoter and alternative transcripts. **a** Schematic representation of the first two exons of *SHOX*. The two promoters, P1 and P2, and the *SHOX* 5′UTR variations are indicated. The variants are numbered relatively to the first nucleotide upstream of the initiation codon (c.-1). The 5′UTR in the mRNA1 is generated from the junction of exon 1 and 2. The shorter mRNA2 originates from P2. **b** Transcript variants of *SHOX*. The two variants are identical at the 5′ end (exons 1–5) but differ in the final exon (6a vs 6b) at the 3′ end.

Nucleotide numbering reflects coding DNA numbering with c.1 corresponding to the first nucleotide of the translation initiation codon. The 5′UTR variants were numbered relative to the first nucleotide upstream of the initiation codon, which was designated c.–1. The genomic reference sequence used for *SHOX* was NG_009385.2, GRCh37/hg19 assembly. Transcript references NM_000451.3 and NM_006883.2 were used for the transcript variants SHOXa and SHOXb, respectively. The two transcripts, identical at the 5′ end (exons 1–5), differ in the final exon (6a vs 6b) at the 3′ end (Fig. 2b).

### Database submission

The variants were submitted to Leiden Open Variation Database^31^ https://databases.lovd.nl/shared (individual IDs: 00295610, 00295612, 00295616, 00295619, 00295622 and 00295613).

### In silico analysis

Splicing regulatory sequences in *SHOX* were predicted using the computational tool Human Splicing Finder version 3.1 [25] (http://www.umd.be/HSF/). The potential miRNA target sites were searched in the *SHOX* 5′UTR using miRWalk 2.0 [26] (http://mirwalk.umm.uni-heidelberg.de/). VarSome [27] annotation tool (www.varsome.com) was used to predict the pathogenicity of the identified variants according to the ACMG guidelines [28], classifying it as one of “pathogenic”, “likely pathogenic,” “likely benign,” “benign” or “uncertain significance.”

### Luciferase assay

#### PGL3 constructs

Luciferase reporter gene expression vectors were prepared according to the modified version of the protocol described in a previous paper [29]. Briefly, the 298 bp region upstream the AUG was PCR amplified using the following primers Shox_5utr_Forward (KpnI), 5′-GTAATAGGTACCAGGTGTACGGACGCCAAACAG-3′ and Shox_5utr_ Reverse (NcoI), 5′-GCCGTGAGCTCTTCCATGGCT-3′. PCR fragments were digested with KpnI and NcoI respectively and cloned into KpnI/NcoI restricted pGL3-basic. The *SHOX* 5′-UTR (from c.-1 to c.-298) was cloned immediately upstream of the Firefly cDNA. The pGL3-basic construct bearing wild-type 5′-UTR fragment was used as the template into which the variants c.-58G > T, c.-55C > T, c.-51G > A, c.-19G > A and c.-9del were introduced by Q5®site-directed mutagenesis kit according to the procedure recommended by the supplier (Supplementary Table 2). DH5a competent cells were transformed with the different constructs and grown on Luria Broth/ampicillin media.

After selecting the correct clones by colony PCR, the plasmid DNA was isolated using Maxiprep kit (Qiagen, Milan, Italy). All the variations in the plasmids were confirmed by bidirectional sequence analysis.

#### Cell culture and luciferase assay

U_2_OS cells were maintained in DMEM High Glucose (Gibco-Life Technologies) supplemented with 10% fetal calf serum and 1% Penicillin/Streptomycin in 5% CO_2_ at 37 °C. A day before transfection 1 × 10^5^ cells were seeded into each well of a 24-well tissue culture plate in 500 μl CGM (Complete Growth Medium). The wells were previously treated with 1:10 dilution Poly-L-lysine solution (Sigma Aldrich) to allow the cells to completely adhere to the plate surface. At 70–90% confluency, cells were transfected with wild-type or mutagenized reporter plasmid constructs and internal control pRL-TK constructs (expressing renilla luciferase gene) with Lipofectamine 2000 transfection reagent (Life Technologies).

Two days after transfection, growth media were removed, and cells were washed gently with phosphate buffered saline. Passive lysis buffer (Promega, Madison, WI) 100 µl/well was added and with gentle rocking for 15 min at room temperature cell lysates were harvested for DLR assay. The activities of firefly and Renilla luciferase were measured using the dual-luciferase reporter assay system (Dual-Glo Luciferase Assay System, Promega, Madison, WI, USA) according to the manual of the manufacturer. For each luminescence reading, after injector dispensing assay reagents into each well, there would be a 2-s pre-measurement delay, followed by a 10-s measurement period. In total, 10 µl of cell lysate were transferred in white opaque 96-well plate. The luminescence obtained for the mutated and wild-type constructs were normalized with the internal control Renilla luciferase signal and the activity of the mutated constructs was reported as percentage with respect to the wild-type. Each experiment was performed in triplicate, and three independent experiments were performed. Quantitative data of the reporter gene assay are calculated as mean ± SEM. Student’s *t* test was used to determine significant differences of each mutated construct compared with the wild-type construct.

#### Quantification of luciferase mRNA expression by real-time PCR

U_2_OS cells were seeded into six-well plates and transfected at 70–80% confluence with 2 µg of the pGL3-based luciferase vectors containing the wild-type or the mutagenized *SHOX* 5′-UTR. Twenty-four hours after transfection, cells were harvested and washed once with phosphate buffer saline. Total RNA was extracted using the QIAGEN RNA Mini Kit (Qiagen, Milan, Italy). Purity and concentration of RNA and genomic DNA were evaluated using a NanoDrop ND1000 spectrophotometer. cDNA was generated from 1 µg of RNA by using the GoScript™ Reverse Transcriptase (Promega, Milan, Italy). Real-time PCR was performed using a cDNA aliquot equivalent to 50 ng of converted RNA using a CFX-96 Real-Time System thermal cycler (BioRad,) and the GoTaq® qPCR Master Mix (Promega, Milan, Italy). Relative mRNA quantification was obtained using the ΔCt method, considering the efficiency of cDNA synthesis by the quantification of the Beta2Microglobulin (β2M) housekeeping gene from the same cDNA.

### mRNA splicing in vitro assay

The pSPL3 vector contains a small artificial gene composed of an SV40 promoter, an exon-intron-exon sequence with functional splice donor and acceptor sites, and a late polyadenylation signal. Within the single intron a multiple cloning site is located, into which a genomic fragment of interest is inserted to create a mini-gene expression construct. Fragments carrying the wild-type or mutant *SHOX* exon 2 flanked by 123 bp of the 3′region intron 1 and 168 bp of the 5′region of intron 2 of *SHOX* were amplified (Primers in supplementary Table 3) and cloned into pSPL3 between the exons SD (Splice Donor) and SA (Splice acceptor) using SacI and BamHI restriction sites (Fig. 4).

The U2O-S cells (3 × 10^5^) were seeded in a six-well culture plate and incubated at 37 °C in a 5% CO_2_ atmosphere in Dulbecco’s modified Eagle medium supplemented with 10% fetal bovine serum (Gibco-BRL, Carlsbad, CA, USA). On the following day, 2 μg of wild-type or mutant vectors were transfected using Lipofectamin 2000 transfection reagent (Life Technologies). The culture medium was changed after 4 h. After 48 h, the cells were harvested and total RNA was isolated using the QIAGEN RNA mini kit (QIAGEN). cDNA was synthesized from 1 µg of RNA by the High Capacity cDNA Reverse Transcription kit (Applied Biosystems), according to the manufacturer’s instructions. Using vector exon-specific primers, cDNAs produced from the mini-gene constructs were specifically PCR amplified and Sanger sequenced.

## Results

### Characterization of *SHOX* 5′UTR variants

During the routine diagnostic testing for the presence of *SHOX* alterations in LWD/ISS, 10 patients belonging to 9 families were identified carrying 5 different rare variants in the 5′UTR within the *SHOX* promoter P_2_ (c.-58G > T, c.-55C > T, c.-51G > A, c.-19G > A, and c.-9del; Fig. 2, Table 2). All the variants, but c.-58G > T, were already reported in the genome aggregation database (gnomAD) with low (c.-55C > T and c.-9del) or very low frequency (c.-51G > A and c.-19G > A). Interestingly, c.-19G > A represents a recurrent variation in our cohort of patients, as it was detected in 4 individuals from 3 unrelated pedigrees out of 1036 tested families. An alternative allele at the same site, namely, c.-19G > C, is also reported in gnomAD with higher frequency (MAF 0.0007).

**Table 2:** *SHOX* 5′UTR variants identified in Italian cohort of ISS patients.

Variant	dbSNP^a^	gnomAD frequency^b^	Present study		LOVD *SHOX* mutation database^c^	VarSome manually adjusted (ACMG tags)
			Patients	Controls		
c.-58G > T	–	–	MAF = 0.0004 1/2072	0/1518	MAF = 0.0004 1/2072	Uncertain Significance PM2, BP4
c.-55C > T	rs772910213	0.002742 (86/31364)	MAF = 0.0014 3/2072	MAF = 0.0039 (6/1518)	MAF = 0.0066 (12/1818)	Benign BS1, BS2, BP4, BP6
c.-51G > A	rs1382937426	0.000004 (1/247626)	MAF = 0.0004 1/2072	0/1518	MAF = 0.0004 1/2072	Likely Pathogenic PS3, PP1, PM2
c.-19G > A	rs201157428^d^	0.000008 (2/248958)	MAF = 0.0014 3/2072	0/1518	MAF = 0.0014 3/2072	Likely Pathogenic PVS1, PS3, PM2, PP1
c.-9del^e^	rs778186580	0.000417 (117/280842)	MAF = 0.0004 1/2072	0/1518	MAF = 0.00069 (4/11568)	Likely Benign PS3, BS2, BP4

Note: Tags for classifying Variants are those according the American College of Medical Genetics and Genomics (ACMG). The analysis was carried out by the VarSome web platform using manually adjusted criteria. The genomic reference sequence used for *SHOX* was NG_009385.2, the Human Chromosome X, GRCh37/hg19 assembly. Transcript references NM_000451.3 and NM_006883.2 were used for the transcript variants SHOXa and SHOXb, respectively. ACMG Tags: PM2- Absent in population databases, BP4- Multiple lines of computational evidence suggest no impact on gene /gene product, BS1- MAF is too high for disorder, BS2- observation in controls inconsistent with disease penetrance, BP6- Reputable source w/out shared data = benign, PS3- Well-established functional studies show a deleterious effect, PP1- Cosegregation with disease in multiple affected family members in a gene definitively known to cause the disease, PVS1- null variant (nonsense, frameshift, canonical ±1 or 2 splice sites, initiation codon, single or multiexon deletion) in a gene where LOF is a known mechanism of disease.

^a^https://www.ncbi.nlm.nih.gov/snp

^b^https://gnomad.broadinstitute.org

^c^https://databases.lovd.nl/shared/genes/SHOX; c.-58G>T, -51G>A, -19G>A were inserted in the database from the present study

^d^This ID in gnomAD corresponds to both c.-19G > A and c.-19G > C.

^e^c.-9del is also documented as c.-10del in some databases.

Due to incomplete penetrance of *SHOX* alterations, a low allele frequency in the gnomAD is not unexpected since their presence does not necessarily rule out a pathogenic role. On the other hand, the height of the individuals in gnomAD is not reported and it is not unlikely that short stature individuals might be included. To assess the actual frequency of the 5′UTR variants in a control population, *SHOX* exon 2 was sequenced in 759 normal stature Italian subjects matched for sex and geographical origin. Only the c.-55C > T variant was identified with a frequency similar to that reported in the SNP databases confirming that it represents a polymorphism. The other four variants were not detected in the control sample (Table 2) and although this result is not statistically significant (two-tailed Fisher’s exact test) it confirms the rarity of these variations. In silico analysis was performed to search for putative miRNA-binding site within the *SHOX* 5′UTR including the here described variants. No miRNA target site was modified by the presence of these variations.

The four variants with possible pathogenic significance, namely c.-58G > T, c.-51G > A, c.-19G > A, and c.-9del were carried by 7 patients (Table 1, Fig. 1) presenting either with severe short stature (#2, #5, #6) and/or body disproportions (#1, #4a) in the presence of other clinical signs, such as cubitus valgus (#2) and muscular hypertrophy (#4a, #4b, 5).

The genetic analysis was extended to the relatives of the patients, revealing that all the variants were inherited from one of the two parents. In four out of six cases, the height of the transmitting parent was in the low range of the height distribution curve between −1.65 SDS and −2.2 SDS (Supplementary Table 1).

### Functional analysis of 5′UTR variants

#### Role of the exon 2 variants on gene expression

**Fig. 3:**
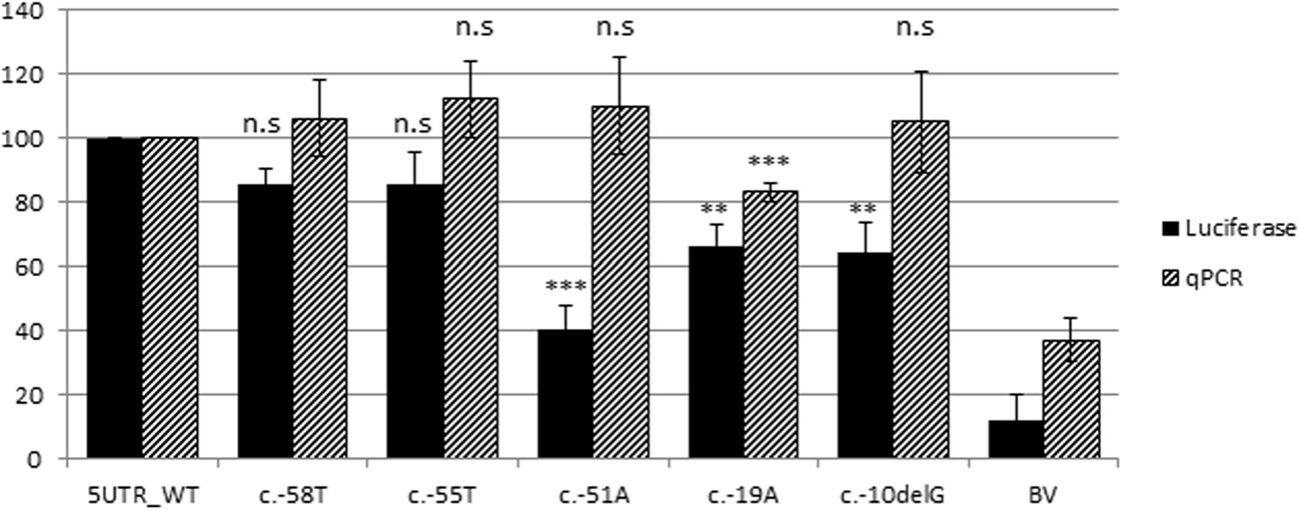
Luciferase activity measured after transient transfections of pGL3 vectors containing the SHOX-5′UTR variations. Activity measured as relative light units (RLUs) is the mean of at least triplicate assays and is presented as a percentage. RLUs were normalized and compared with activity of the corresponding WT construct (black bars). Luciferase mRNA expression normalized to the relative cDNA synthesis efficiency as revealed by the amplification of β2M housekeeping mRNA (striped bars). Significance levels are indicated as follows: ns *P* > 0.05, * *P* ≤ 0.05, **P ≤ 0.01, ***P ≤ 0.001. BV basic vector.

To evaluate whether the P_2_ variant might affect transcription, an expression plasmid harboring the region between c.-298 and c.-1 of *SHOX* exon 2 (containing P_2_ and the 5′UTR), placed upstream of the firefly luciferase reporter gene (pGL3-*SHOX*), was created. The transcription efficiency of the plasmid carrying the five variants, including c.-55C > T as a potential negative control, was measured and compared with the wild-type by transfecting a U_2_OS cell line. Plasmids carrying c.-55C > T and c.-58G > T did not show any significant reduction in the level of luciferase activity compared with the wild-type (Fig. 3). This result further confirms that c.-55C > T is a benign polymorphism (Fig. 3). In contrast, the variants c.-51G > A, c.-19G > A, and c.-9del showed significantly reduced luciferase activity of 60% (*p* = 0.00967), 35% (*p* = 0.02147), and 40% (*p* = 0.0262), respectively.

To investigate whether the impact of the 5′UTR variants on reporter activity could be related to transcriptional or post-transcriptional effects, firefly luciferase mRNA was quantified by real-time PCR. Only the construct bearing c.-19G > A showed a significant reduction in mRNA expression (Fig. 3), suggesting that this variant might have an effect at the transcriptional level, whereas the others, c.-51G > A and c.-9del, which showed no reduction in relative mRNA levels, might interfere with the correct *SHOX* expression, mainly at the post-transcriptional level (Fig. 3).

### Role of c.–19 G > A on mRNA splicing

**Fig. 4:**
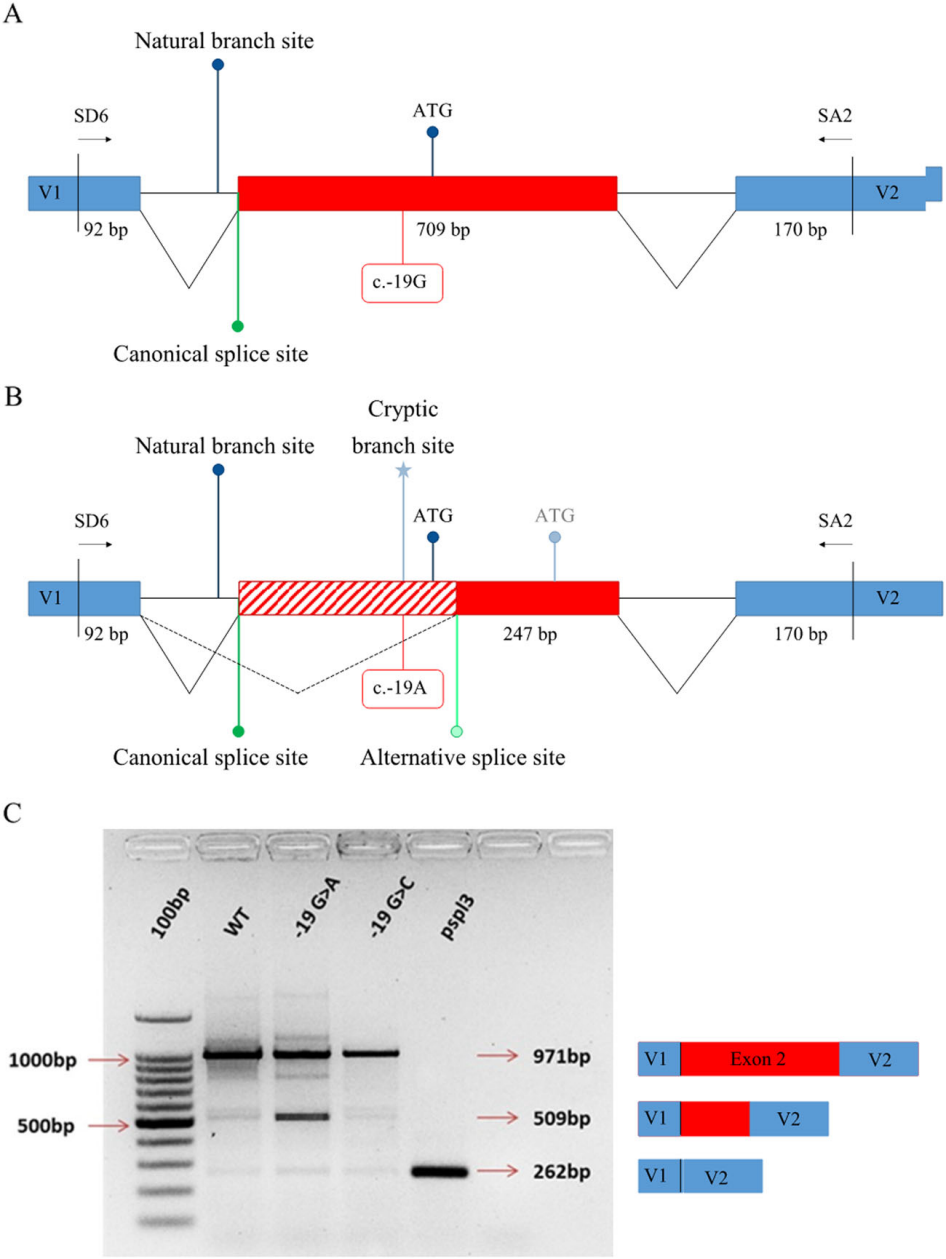
Exon trapping assay. **a** Wild-type construct (c.-19G): pSPL3 vector exons V1 and V2 are indicated. Vector exon-specific primers are indicated by half-arrows as SD6 and SA2. **b** Mutated construct (c.-19A): the cryptic branch point is indicated by an asterisk (*). The alternative splicing pattern is indicated by dashed lines. The creation of a novel branch point result in an alternative splicing between exon 1 and exon 2, removing the ATG start codon. The first available ATG in the mutated allele is indicated, that leads to a termination codon 7 amino acid downstream. **c** Gel electrophoresis of RT-PCR products from transfected U2O-S cells. The Wild-type (WT) construct produced a band of 971 bp corresponding to the correct usage of the canonical splice sites (lane 2). The construct bearing c.-19A showed an additional band of 509 bp corresponding to the aberrant mRNA (lane 3) originating by the skipping of part of exon 2. The construct carrying the c.-19C produced a normal transcript (lane 4) as well as WT (lane 2). Spliced product from cells transfected with vector containing no inserted gDNA is indicated with a band size of 262 bp (lane 5). Wild-type and mutant transcript contents were confirmed by Sanger sequencing and are depicted to the right of the gel image.

Exon 2 containing the P_2_ promoter and the 5′UTR of mRNA2 is included within the longer transcript, mRNA1, generated from P_1_ (Fig. 2), which contains a 5′UTR resulting from the junction of exon 1 and exon 2 (Fig. 2). Interestingly, an in silico analysis performed through the online tool Human Splicing Finder (http://www.umd.be/HSF/) predicted that the variant c.-19G > A alters the correct splicing between exons 1 and 2 in mRNA1 by creating a consensus sequence for a novel putative branch point within exon 2. The consequence might be the usage of an alternative cryptic acceptor splice site located downstream of the canonical AUG (Fig. 4). This putative branch point is predicted by the software with a consensus value score (CV = 93.9) that is significantly higher than the CV score of the natural branch point within intron 1 (CV = 78.35).

To test this hypothesis, an in vitro analysis was performed using a minigene splicing assay based on the pSPL3 exon trapping vector. The in vitro splicing assay followed by RT-PCR and sequencing of the PCR products revealed that c.-19G > A causes aberrant splicing. The transcript originating from the wild-type construct bearing c.-19G corresponded to a single product of 971 bp resulting from the correct splicing of exon 2 (SHOX transcript NM_000451.3). Conversely, the plasmid carrying c.-19A, in addition to the wild-type 971 bp fragment, produced a smaller product of 509 bp. The additional 509 bp band is originated by the creation of the strong branch site at c.-19 that promotes the usage of the cryptic acceptor splice site located 27 bp downstream of the canonical AUG start codon (Fig. 4). As a control, a construct carrying the alternative polymorphic allele c.-19C (not predicted in silico to create a novel branch point) was prepared and assayed for the its effect on exon 2 splicing (Fig. 4). No aberrant spliced product was observed thus confirming that the negative effect is exerted by the presence of the nucleotide A at position c.-19.

## Discussion

Functional studies for determining the clinical significance of variants in coding regions are widely described. In contrast, it is harder to draw solid conclusions and communicate information during genetic counseling for carriers of variants in the noncoding regions, with some exception, such as intronic splicing variants whose relation to the disease may be clearly demonstrated [9]. In the case of short stature patients, assessing the pathogenicity of the 5′UTR *SHOX* variations is critical not only for counseling but also for treating the patients with an effective GH replacement therapy [30].

During the *SHOX* diagnostic routine, we identified 4 potentially relevant variations within the *SHOX* 5′UTR in 7 patients belonging to 6 families that represented 5.6% (6/107) of the probands carrying *SHOX* alterations. Based on the current knowledge, it was not possible to attribute a pathogenic role to these variations that were thus classified as VUS.

Analysis of the family members of the patients (Fig. 1) revealed that, in four cases out of six, these variants were inherited from a parent who was either short stature (SDS <-2) or in the lower part of the curve. However, the incomplete penetrance and the small dimension of the families render the segregation analysis of limited utility. It is very difficult to assess the pathogenicity of a variant by segregation analysis in *SHOX*-related disorders unless carriers either clearly manifest the clinical signs or the variant arose de novo.

Thus, the establishment of functional studies is of crucial importance to gain insight into the significance of these noncoding variants. The 5′UTRs are the sites that serve ribosomes for scanning the mRNA for a suitable translational start codon at which the translation initiation complex can be assembled. This scanning can be influenced by several cis elements, including mRNA secondary structures, the presence of upstream initiation codons and in frame stop codons that act as cis regulatory elements [31, 32]. Previous studies highlighted that pathogenic variations in the 5′UTR can alter the amounts of essential proteins and may be causative of human diseases either by creating new initiation codons [33] or by affecting splicing [34] or by post-transcriptional modification of RNA (secondary structure and mRNA stability) or by alteration of translational efficiency [35–37]. Our experimental data showed that variations within the *SHOX* 5′UTR might influence gene expression and correct splicing and might thus lead to insufficiency of the SHOX protein and thus to related disorders.

The luciferase activity displayed by c.-51G > A, c.-19G > A, and c.-9del was reduced by 60, 35, and 40% under the in vitro experimental condition. In vivo these variations were carried at the heterozygous state and the overall impact on SHOX expression might be different with respect to what observed in vitro. However several previous studies demonstrated that 5′UTR variants that did not completely abolish the luciferase activity were causative of disorders [38–40]. On the other hand most of the *SHOX* pathogenic alterations affecting the CNEs regulatory regions are present at the heterozygous state showing an extreme variable expressivity, that might be the consequence of different level of expression in different individuals of the mutated allele that not is completely silenced.

To test whether the reduced luciferase activity was the consequence of a lower transcription efficiency or a decreased translation, the luciferase mRNA levels were quantified by real-time PCR. The plasmid carrying c.-51G > A and c.-9del generated mRNA levels similar to the wild-type, thus suggesting that the reduced luciferase activity (Fig. 3) could be attributable to post-transcriptional rather than transcriptional effects, leading to a lower amount of the translated protein (Luciferase) for example, through mRNA secondary structure modification determined by these variants. These 5′UTR variants (c.-51G > A and c.-9del) that are predicted to decrease the expression by 30% and 20%, respectively, in their heterozygous state can be considered as mild effect alleles.

The variant c.-58G > T did not show any functional effect, it was inherited from a normal height father and although absent in the gnomAD and in the 1518 alleles from our cohort of normal stature individuals (Table 2), it might represent a very rare likely benign variant.

Based on the frequency reported in gnomAD (Table 2) and the analysis performed using VarSome, the variant c.-9del might be classified as a polymorphic variation even if absent in the here analyzed control cohort. However, considering its in vitro effect, the pathogenicity cannot still be definitively ruled out and it remains a variation of uncertain significance.

Although the pedigree is too small to draw solid conclusion, the patients carrying c.-51G > A showed severe short stature (-2.9 SDS) and inherited the variant from a short stature father (-2.3 SDS), which is suggestive of cosegregation of the variant and the phenotype. By combining this information with the experimental results obtained in vitro, we can now refer c.-51G > A as a variant of mild effect which might contribute to affect SHOX expression.

Although the variant c.-19G > A showed a mild influence on the transcriptional activity (65% compared with the wild-type), we clearly demonstrated that the main impact of this variant is on mRNA splicing through the creation of a novel branch site that leads to an aberrant splicing between exon 1 and 2, removing part of the 5′UTR of mRNA1 and the AUG start codon (Fig. 4). The usage of alternative AUGs located downstream of the canonical start codon is predicted to yield proteins with altered reading frames resulting in a premature stop codon (Fig. 4). The co-occurrence in the transcript from construct bearing the c.-19G > A of the two bands of 971 and 509 bp, corresponding to the wild-type and aberrant splicing respectively, suggests that the both the canonical and the novel branch sites are used in vitro. As in other examples of disease causing splicing variants the activation of a cryptic branch site leading to an aberrant mRNA does not exclude the persistence of the wild-type mRNA as the canonical branch site, albeit with a lower consensus value is still intact [41]. Different is the case of variants that eliminates the canonical branch site with the consequent abolition of the normal mRNA and the presence of total aberrant mRNA [42]. However, the physiological amount of the wild-type protein and of the wild-type/mutant mRNA ratio in patients carrying c.-19G > A are not exactly predictable as they depend on many factors in vivo during the chondrocyte proliferation and maturation that regulate for example the usage of the two different promoters and the translation of mRNAs carrying premature stop codons that might cause a slowdown in the translational machinery. Nevertheless, it is likely that the global amount of SHOX functioning protein is altered leading to SHOX insufficiency. As the SHOX expression is driven by two promoters P1 and P2 (Fig. 1), the two types of mRNAs are translated with different efficiency, thereby contributing to the fine-tuned regulation of SHOX expression. By combining luciferase assay results and the in vitro splicing assay, it might be hypothesised that c.-19G > A exert a combined effect on the translation of mRNA2 and the transcription of mRNA1.

Based on the functional results, population frequency and screening of a normal stature control population, it is now possible to attribute a likely pathogenic significance to at least the c.-51 G > A and c.-19G > A variations detected in 4 pedigrees (5 patients) out of 1036 (0.0038) short stature unrelated individuals as they exhibited a functional significance in vitro, were absent in our control population and showed a frequency <0.001% in the public database. The presence of these two variations in gnomAD can be explained by the prevalence of short stature individuals (-2 SDS) that represent 3% of the population. Considering that c.-19G > A and c.-51 G > A represent 0.38% of the variants identified in our cohort of short stature individuals it is not unexpected that these variations show a frequency in the gnomAD database of 0.000008 and 0.000004, respectively.

In conclusion, this study allowed us to reclassify some of the new variants of uncertain significance identified during diagnostic screening as likely pathogenic. In particular, variants in the 5′UTR might affect the splicing in a subtle way and might represent an important source of SHOX haploinsufficiency.

## Supplementary information

Supplementary Information

## References

[1] Rao E, Weiss B, Fukami M, Rump A, Niesler B, Mertz A (1997). Pseudoautosomal deletions encompassing a novel homeobox gene cause growth failure in idiopathic short stature and Turner syndrome. Nat Genet.

[2] Sabherwal N, Bangs F, Röth R, Weiss B, Jantz K, Tiecke E (2007). Long-range conserved non-coding SHOX sequences regulate expression in developing chicken limb and are associated with short stature phenotypes in human patients. Hum Mol Genet.

[3] Chen J, Wildhardt G, Zhong Z, Röth R, Weiss B, Steinberger D (2009). Enhancer deletions of the SHOX gene as a frequent cause of short stature: the essential role of a 250 kb downstream regulatory domain. J Med Genet.

[4] Leka SK, Kitsiou-Tzeli S, Kalpini-Mavrou A, Kanavakis E (2006). Short stature and dysmorphology associated with defects in the SHOX gene. Hormones.

[5] Jorge AA, Souza SC, Nishi MY, Billerbeck AE, Libório DC, Kim CA (2007). SHOX mutations in idiopathic short stature and Leri-Weill dyschondrosteosis: frequency and phenotypic variability. Clin Endocrinol (Oxf).

[6] Rappold GA, Fukami M, Niesler B, Schiller S, Zumkeller W, Bettendorf M (2002). Deletions of the homeobox gene SHOX (short stature homeobox) are an important cause of growth failure in children with short stature. J Clin Endocrinol Metab.

[7] Rappold G, Blum WF, Shavrikova EP, Crowe BJ, Roeth R, Quigley CA (2007). Genotypes and phenotypes in children with short stature: clinical indicators of SHOX haploinsufficiency. J Med Genet.

[8] Stuppia L, Calabrese G, Gatta V, Pintor S, Morizio E, Fantasia D (2003). SHOX mutations detected by FISH and direct sequencing in patients with short stature. J Med Genet.

[9] Hirschfeldova K, Solc R, Baxova A, Zapletalova J, Kebrdlova V, Gaillyova R (2012). SHOX gene defects and selected dysmorphic signs in patients of idiopathic short stature and Léri-Weill dyschondrosteosis. Gene.

[10] Genoni G, Monzani A, Castagno M, Ricotti R, Rapa A, Petri A (2018). Improving clinical diagnosis in SHOX deficiency: the importance of growth velocity. Pediatr Res.

[11] Marchini A, Ogata T, Rappold GA (2016). A track record on SHOX: from basic research to complex models and therapy. Endocr Rev.

[12] Bertorelli R, Capone L, Ambrosetti F, Garavelli L, Varriale L, Mazza V (2007). The homozygous deletion of the 3’ enhancer of the SHOX gene causes Langer mesomelic dysplasia. Clin Genet.

[13] Bunyan DJ, Baker KR, Harvey JF, Thomas NS (2013). Diagnostic screening identifies a wide range of mutations involving the SHOX gene, including a common 47.5 kb deletion 160 kb downstream with a variable phenotypic effect. Am J Med Genet A.

[14] Gatta V, Antonucci I, Morizio E, Palka C, Fischetto R, Mokini V (2007). Identification and characterization of different SHOX gene deletions in patients with Leri-Weill dyschondrosteosys by MLPA assay. J Hum Genet.

[15] Benito-Sanz S, Barroso E, Heine-Suñer D, Hisado-Oliva A, Romanelli V, Rosell J (2011). Clinical and molecular evaluation of SHOX/PAR1 duplications in Leri-Weill dyschondrosteosis (LWD) and idiopathic short stature (ISS). J Clin Endocrinol Metab.

[16] Fukami M, Naiki Y, Muroya K, Hamajima T, Soneda S, Horikawa R (2015). Rare pseudoautosomal copy-number variations involving SHOX and/or its flanking regions in individuals with and without short stature. J Hum Genet.

[17] Hirschfeldova K, Solc R (2017). Comparison of SHOX and associated elements duplications distribution between patients (Lėri-Weill dyschondrosteosis/idiopathic short stature) and population sample. Gene.

[18] Monzani A, Babu D, Mellone S, Genoni G, Fanelli A, Prodam F (2019). Co-occurrence of genomic imbalances on Xp22.1 in the SHOX region and 15q25.2 in a girl with short stature, precocious puberty, urogenital malformations and bone anomalies. BMC Med Genomics.

[19] Benito-Sanz S, Aza-Carmona M, Rodríguez-Estevez A, Rica-Etxebarria I, Gracia R, Campos-Barros A (2012). Identification of the first PAR1 deletion encompassing upstream SHOX enhancers in a family with idiopathic short stature. Eur J Hum Genet.

[20] Kant SG, Broekman SJ, de Wit CC, Bos M, Scheltinga SA, Bakker E (2013). Phenotypic characterization of patients with deletions in the 3’-flanking SHOX region. PeerJ.

[21] Blaschke RJ, Töpfer C, Marchini A, Steinbeisser H, Janssen JW, Rappold GA (2003). Transcriptional and translational regulation of the Leri-Weill and Turner syndrome homeobox gene SHOX. J Biol Chem.

[22] Cacciari E, Milani S, Balsamo A, Spada E, Bona G, Cavallo L (2006). Italian cross-sectional growth charts for height, weight and BMI (2 to 20 yr). J Endocrinol Invest.

[23] Bogin B, Varela-Silva MI (2010). Leg length, body proportion, and health: a review with a note on beauty. Int J Environ Res Public Health.

[24] Tanner JM, Whitehouse RH, Cameron N, Marshall WA, Healy WA (1988). H G. Assessment of skeletal maturity and prediction of adult height (TW2 method).

[25] Desmet FO, Hamroun D, Lalande M, Collod-Béroud G, Claustres M, Béroud C (2009). Human splicing finder: an online bioinformatics tool to predict splicing signals. Nucleic Acids Res.

[26] Sticht C, De La Torre C, Parveen A, Gretz N (2018). miRWalk: an online resource for prediction of microRNA binding sites. PLoS One.

[27] Kopanos C, Tsiolkas V, Kouris A, Chapple CE, Albarca Aguilera M, Meyer R (2019). VarSome: the human genomic variant search engine. Bioinformatics.

[28] Richards S, Aziz N, Bale S, Bick D, Das S, Gastier-Foster J (2015). Standards and guidelines for the interpretation of sequence variants: a joint consensus recommendation of the American College of Medical Genetics and Genomics and the Association for Molecular Pathology. Genet Med.

[29] Giordano M, Godi M, Giacopelli F, Lessi M, Mellone S, Paracchini R (2006). A variation in a Pit-1 site in the growth hormone gene (GH1) promoter induces a differential transcriptional activity. Mol Cell Endocrinol.

[30] Dauber A, Rosenfeld RG, Hirschhorn JN (2014). Genetic evaluation of short stature. J Clin Endocrinol Metab.

[31] Araujo PR, Yoon K, Ko D, Smith AD, Qiao M, Suresh U (2012). Before it gets started: regulating translation at the 5’ UTR. Comp Funct Genomics.

[32] Jackson RJ, Hellen CU, Pestova TV (2010). The mechanism of eukaryotic translation initiation and principles of its regulation. Nat Rev Mol Cell Biol.

[33] Semler O, Garbes L, Keupp K, Swan D, Zimmermann K, Becker J (2012). A mutation in the 5’-UTR of IFITM5 creates an in-frame start codon and causes autosomal-dominant osteogenesis imperfecta type V with hyperplastic callus. Am J Hum Genet.

[34] Kramer M, Sponholz C, Slaba M, Wissuwa B, Claus RA, Menzel U (2013). Alternative 5’ untranslated regions are involved in expression regulation of human heme oxygenase-1. PLoS One.

[35] Wang G, Guo X, Floros J (2005). Differences in the translation efficiency and mRNA stability mediated by 5’-UTR splice variants of human SP-A1 and SP-A2 genes. Am J Physiol Lung Cell Mol Physiol.

[36] Cannons AC, Cannon J (2002). The stability of the Chlorella nitrate reductase mRNA is determined by the secondary structure of the 5’-UTR: implications for posttranscriptional regulation of nitrate reductase. Planta.

[37] Hua XJ, Van de Cotte B, Van Montagu M, Verbruggen N (2001). The 5’ untranslated region of the At-P5R gene is involved in both transcriptional and post-transcriptional regulation. Plant J.

[38] Khamis A, Palmen J, Lench N, Taylor A, Badmus E, Leigh S (2015). Functional analysis of four LDLR 5’UTR and promoter variants in patients with familial hypercholesterolaemia. Eur J Hum Genet.

[39] Bisio A, Nasti S, Jordan JJ, Gargiulo S, Pastorino L, Provenzani A (2010). Functional analysis of CDKN2A/p16INK4a 5’-UTR variants predisposing to melanoma. Hum Mol Genet.

[40] Andreotti V, Bisio A, Bressac-de Paillerets B, Harland M, Cabaret O, Newton-Bishop J (2016). The CDKN2A/p16(INK) (4a) 5’UTR sequence and translational regulation: impact of novel variants predisposing to melanoma. Pigment Cell Melanoma Res.

[41] de Boer M, van Leeuwen K, Hauri-Hohl M, Roos D (2019). Activation of cryptic splice sites in three patients with chronic granulomatous disease. Mol Genet Genom Med.

[42] Vivenza D, Guazzarotti L, Godi M, Frasca D, di Natale B, Momigliano-Richiardi P (2006). A novel deletion in the GH1 gene including the IVS3 branch site responsible for autosomal dominant isolated growth hormone deficiency. J Clin Endocrinol Metab.

