# Supplementary material for: Variants in the 5′UTR reduce SHOX expression and contribute to SHOX haploinsufficiency
Source: Eur J Hum Genet. 2020 Jul 9;29(1):110–21. doi: 10.1038/s41431-020-0676-y (PMC7852508; doi:10.1038/s41431-020-0676-y)

**SUPPLEMENTARY INFORMATION**

Supplementary Table 1: Primer sequences for amplification of *SHOX*

| Primer | sequence 5’ → 3’ | Amplified Fragment |
| --- | --- | --- |
| shox_1F | GATCCGGAGATTCGCTTTTC | Exon 1 |
| shox_1R | GCTCACCAAGCTCTGAGCGCAG |  |
| shox_2F | GAGACGCGCGCATCCACCA | Exon 2 |
| shox_2R | GAGGCGCCGAACCCCAGGAG |  |
| shox_3F | CACGTTGCGCAAAACCTC | Exon 3 |
| shox_3R | CGTCCCTCACCCAACCTC |  |
| shox_4-5F | AGTGCTTGGTTCAGCCTCAT | Exon 4-5 |
| shox_4-5R | TTTCTAAGGGCCAGCTGAGA |  |
| shox_6aF | AAGAGGCACGTTGGAGGTTT | Exon 6a |
| shox_6aR | CGGGGTTGAGTGCAGGAC |  |
| shox_6bF | CTCCTCTTCCCGGGTTCAC | Exon 6b |
| shox_6bR | GGTGGTGGGCACCTGTAA |  |

Supplementary Table 2: Primer sequences for site directed mutagenesis

| Primer | sequence 5’ → 3’ |
| --- | --- |
| shox_5utr_ c.-58T_F | Phos – TCGCGGGGAGACGCGCGCATCC |
| shox_5utr_ c.-58T_R | Phos – GGAGAGGACGGCCCGTGCGCGC |
| shox_5utr_ c.-55T_F | Phos – TGGGGAGACGCGCGCATCCACC |
| shox_5utr_ c.-55T_R | Phos – CGCGGAGAGGACGGCCCGTGCG |
| shox_5utr_ c.-51A_F | Phos – AAGACGCGCGCATCCACCAGCC |
| shox_5utr_ c.-51A_R | Phos – CCCGCGCGGAGAGGACGGCCC |
| shox_5utr_c.-19A_F | Phos– ACCAGCCCCGGCCCCAGCCATG |
| shox_5utr_c.-19A_R | Phos– GAGCAGCCGGGGCTGGTGGATG |
| shox_5utr_ c.-9del _F | Phos– GCCCCAGCCATGGAAGACGC |
| shox_5utr_ c.-9del _R | Phos– GGGGCTGGCGAGCAGCCGGG |

Supplementary Table 3: Primer sequences used for exon-trapping assay

| Primer | sequence 5’ → 3’ |
| --- | --- |
| shox_Ex2_SacI_F | ATATATGAGCTCGGCGCTTCCTGGAAAGATC |
| shox_Ex2_BamH1_R | ATATATGGATCCTCCTGCCTTCCTTCCTTTCT |

Supplementary Figures


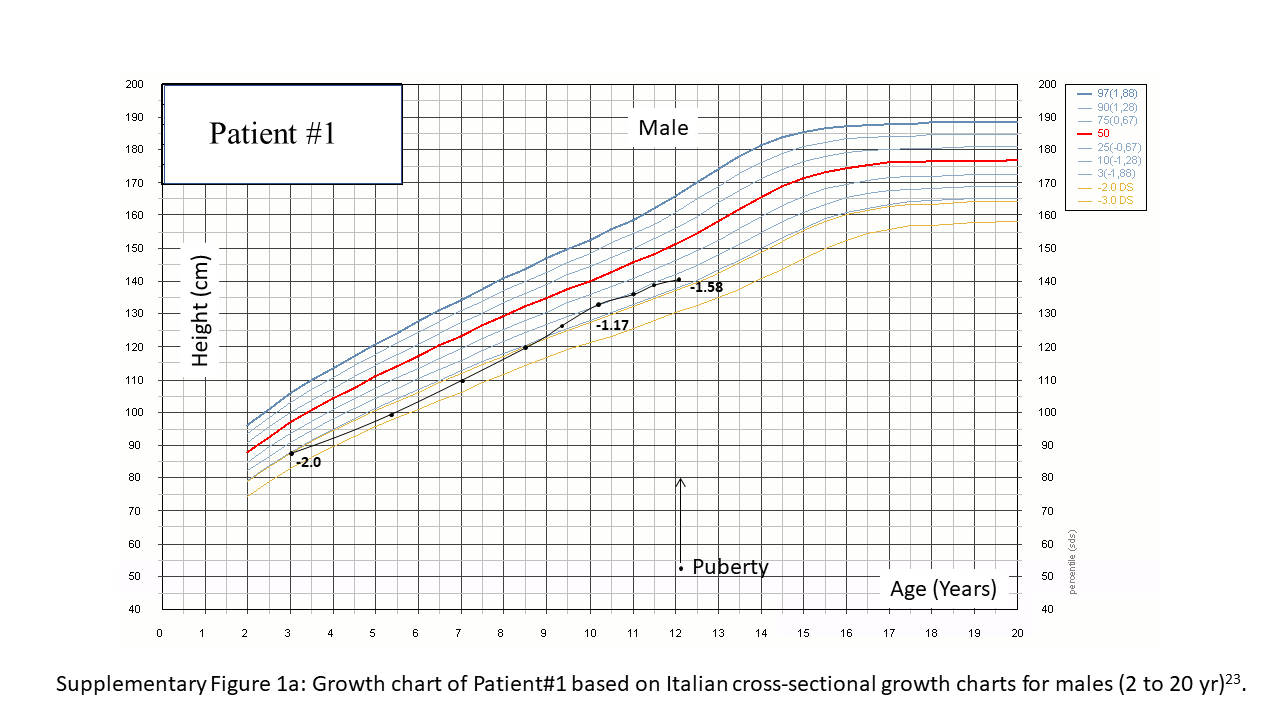


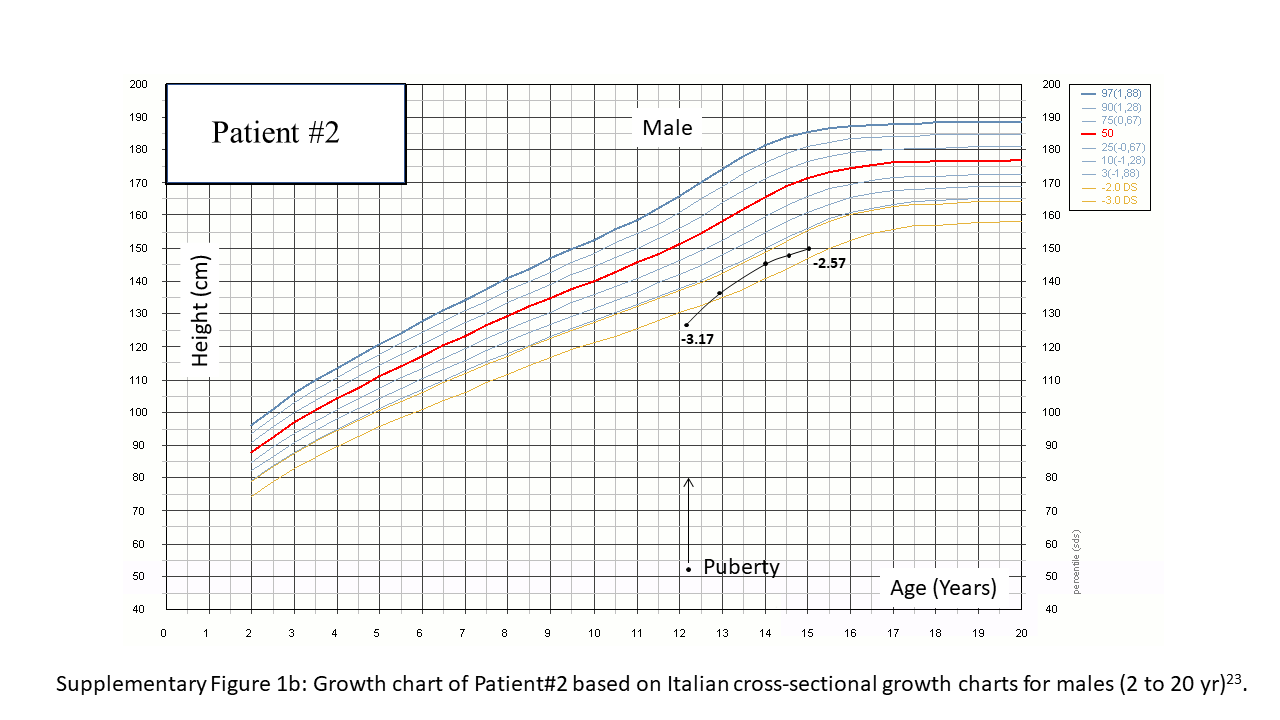


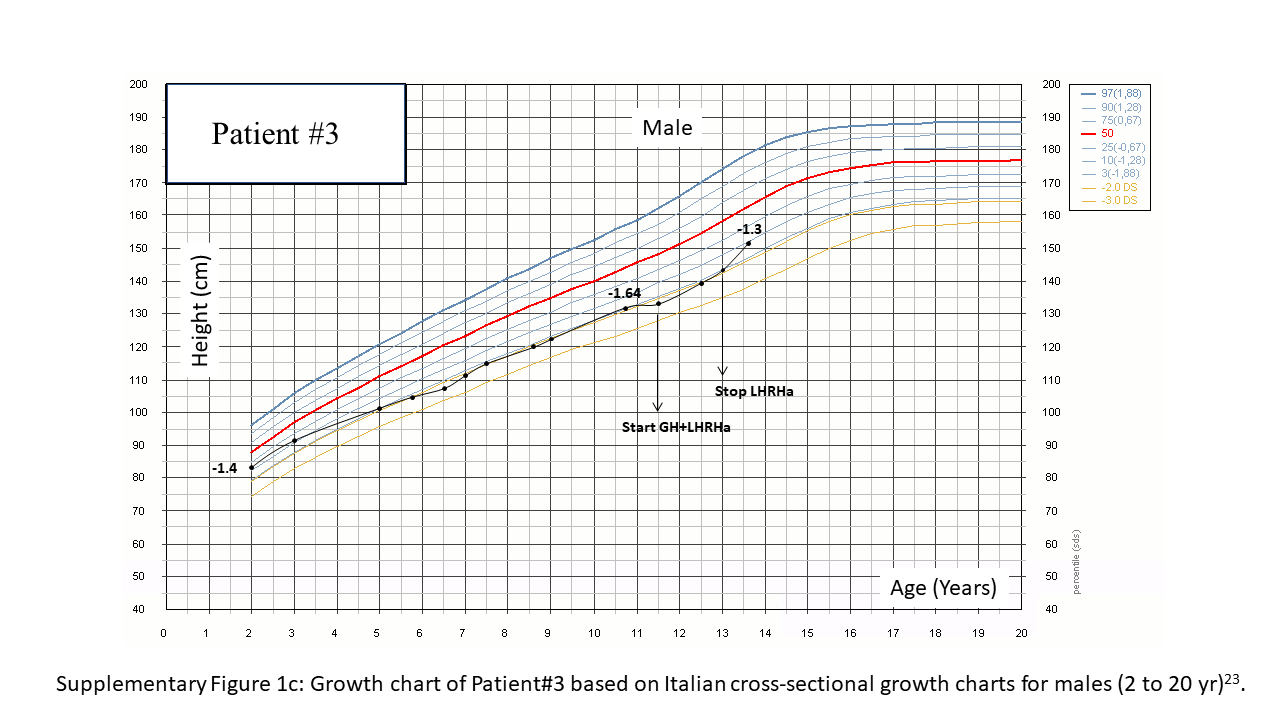


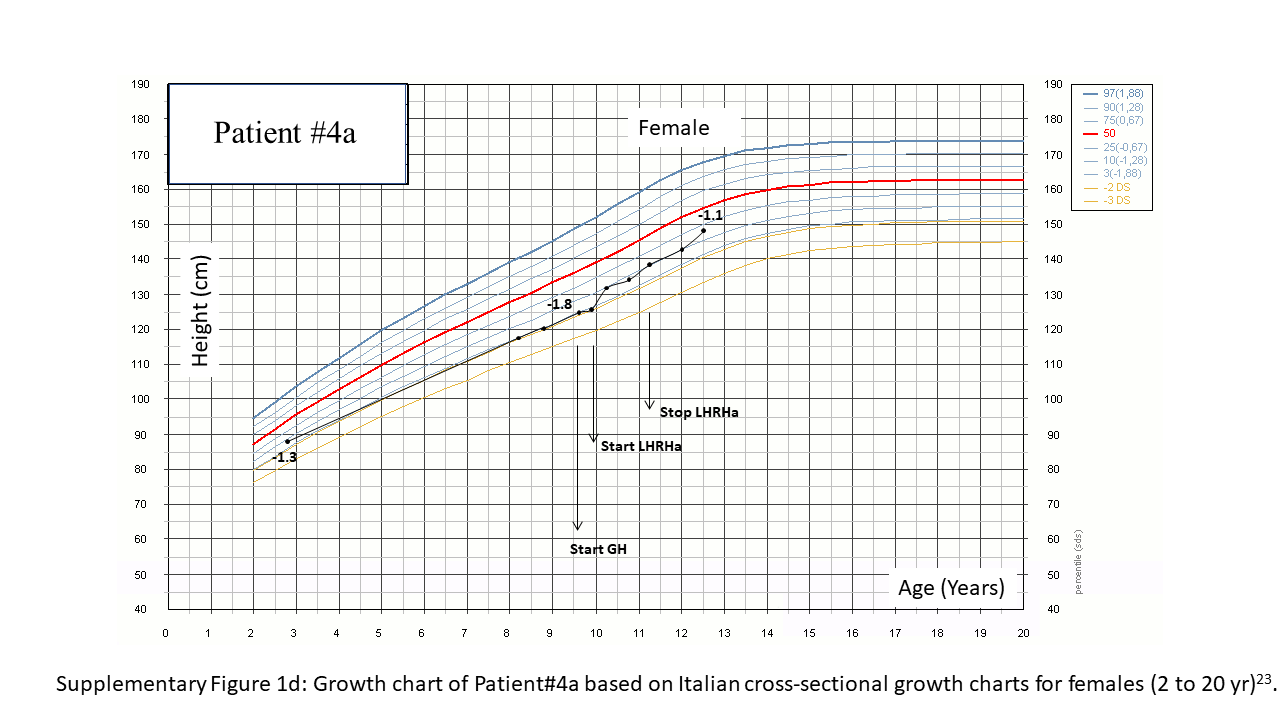


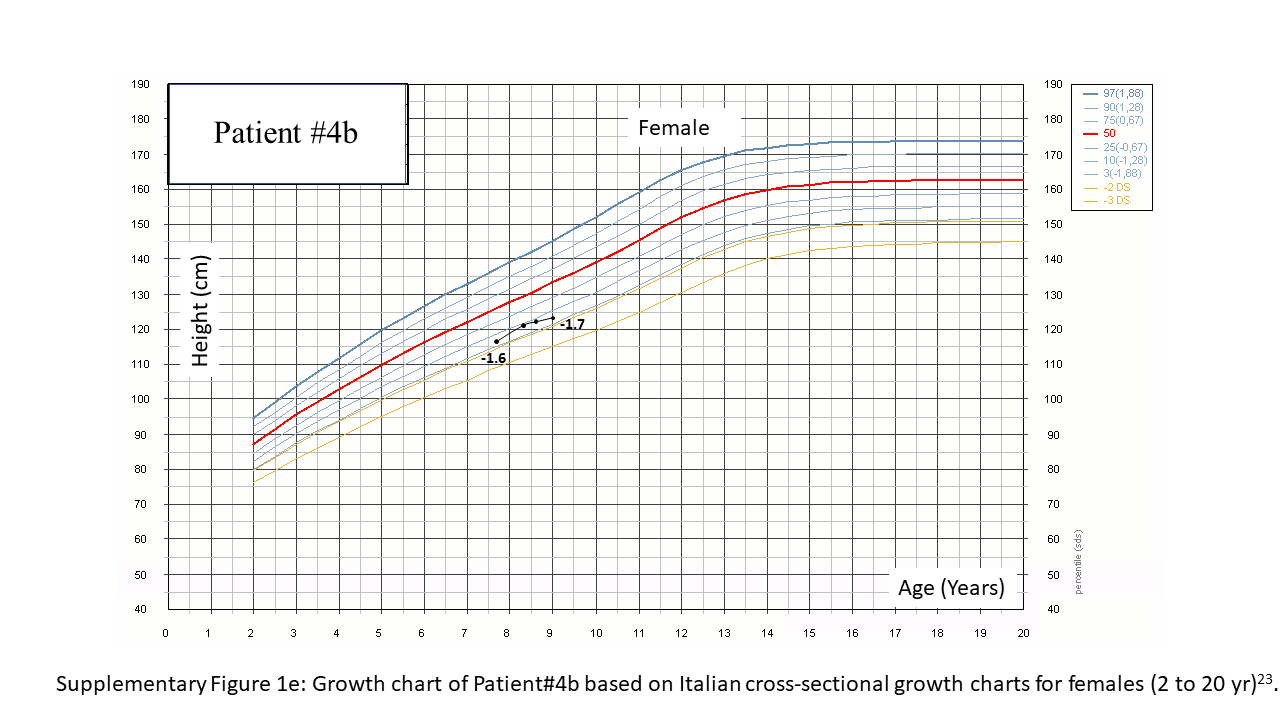


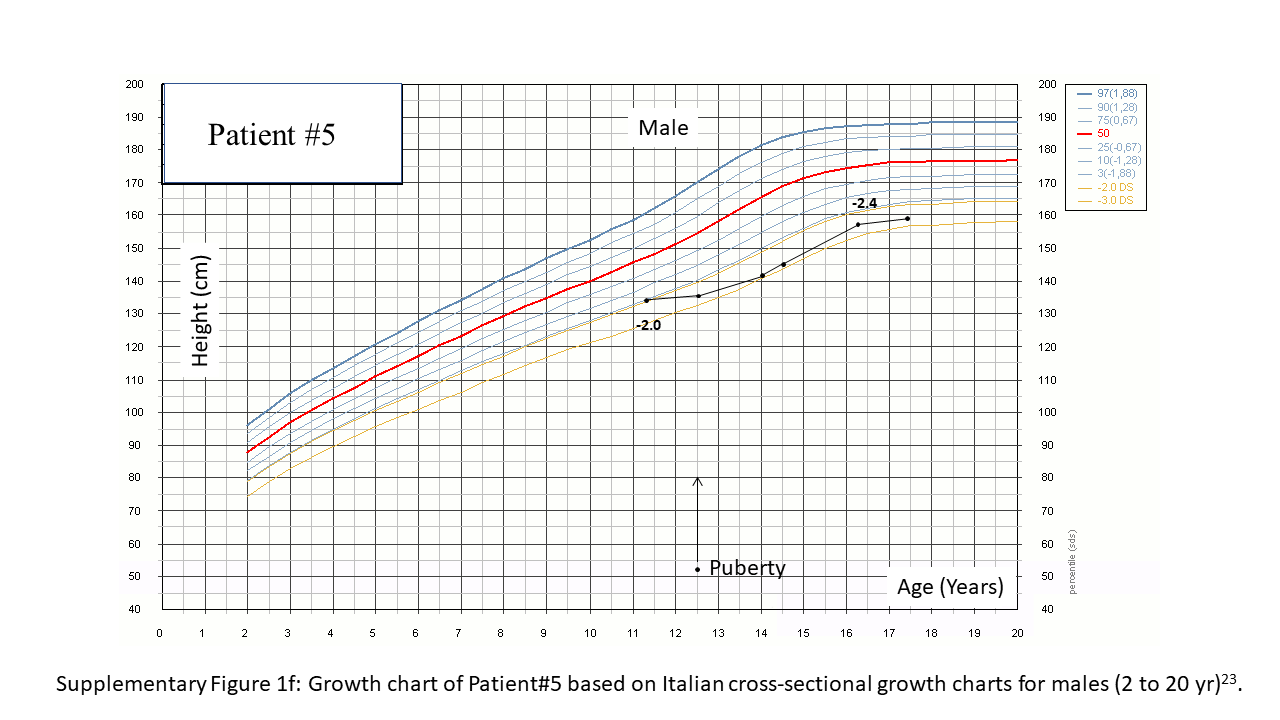


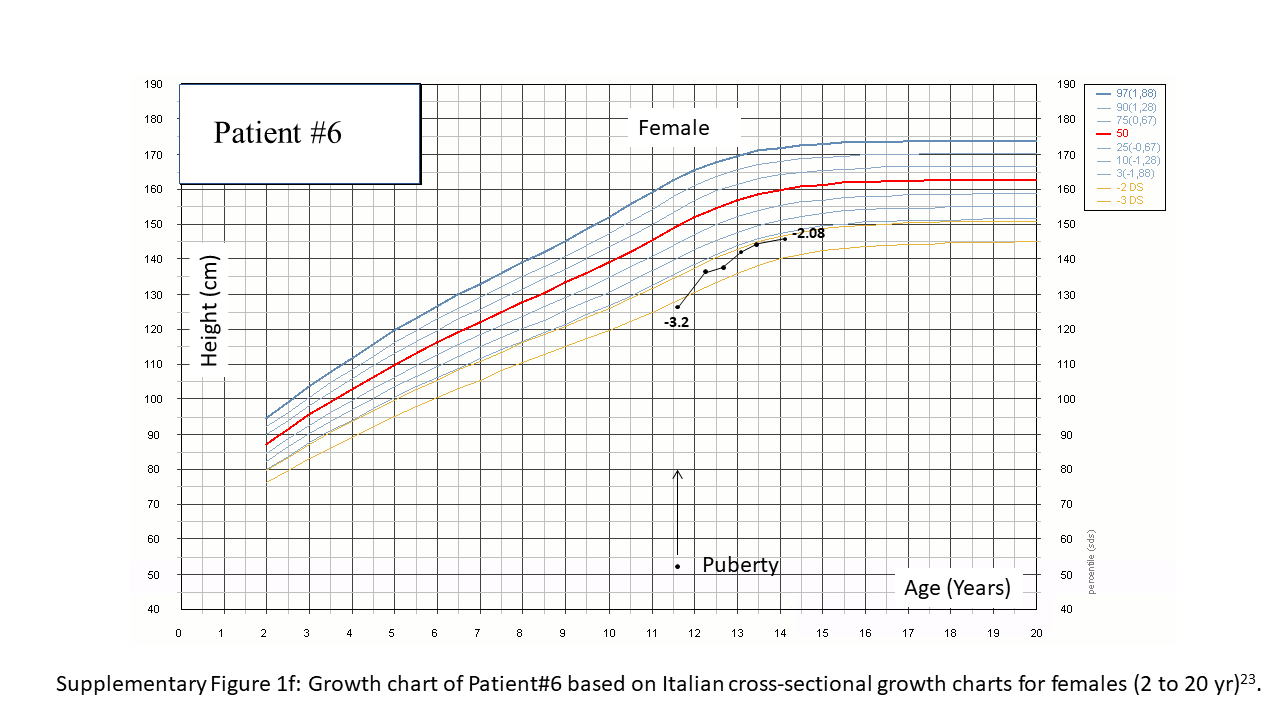

Supplement: Supplementary file 1 — Supplementary Information [file 41431_2020_676_MOESM1_ESM.docx]
